# Development of a TaqMan Probe-Based Quantitative PCR Assay for the Detection of *Cynoglossus semilaevis* Papillomavirus

**DOI:** 10.3390/v18090939

**Published:** 2026-08-27

**Authors:** Xinrui Liu, Menghan Xu, Siyu Yang, Xiang Li, Lei Jia, Ye-Xuan Liu, Shuxia Xue

**Affiliations:** 1 College of Life Science, Tianjin Normal University, Tianjin 300387, China; liuxinruisky@stu.tjnu.edu.cn (X.L.); 15227588715@163.com (M.X.); ysy1638@foxmail.com (S.Y.); 17536051188@163.com (Y.-X.L.); 2 Tianjin Fishery Research Institute, Tianjin 300221, China; ldg1321421@163.com (X.L.); tianjinbohaisuo@163.com (L.J.); 3 Tianjin Key Laboratory of Animal and Plant Resistance, Tianjin Normal University, Tianjin 300387, China

**Keywords:** TaqMan probe-based quantitative PCR, *Cynoglossus semilaevis* papillomavirus, molecular epidemiological characteristics, viral detection

## Abstract

*Cynoglossus semilaevis* papillomavirus (CsPaV) is an emerging viral pathogen associated with high mortality in farmed Chinese tongue soles. A sensitive, specific, and quantitative method is needed for CsPaV surveillance and epidemiological studies. In this study, we designed primers and a probe that targeted the *CsPaV L1* gene. We then established a TaqMan probe-based quantitative PCR (qPCR) assay. The assay detected as few as 5.8 × 10^1^ copies/µL and was 1000-fold more sensitive than conventional PCR. The assay did not cross-react with the other aquatic pathogens tested. The intra-assay coefficients of variation ranged from 0.23% to 0.84%, and the inter-assay coefficients ranged from 0.56% to 1.48%. These results showed that the assay had good repeatability. We used the assay to study the tissue distribution and temporal dynamics of CsPaV after experimental infection. Healthy Chinese tongue soles received an intraperitoneal injection of 300 µL of inoculum containing 3.66 × 10^5^ CsPaV copies/µL. Three fish were sampled at 1, 2, 3, 5, and 7 days post-infection. CsPaV DNA was detected in all tissues examined. The kidney had the highest viral DNA load, which reached 2.47 × 10^7^ copies/mg at 7 days post-infection. Among 176 clinically diseased Chinese tongue soles collected between 2023 and 2026, 80.68% tested positive for CsPaV. CsPaV DNA was also detected in several other aquatic species. These findings provide a sensitive tool for CsPaV surveillance and molecular epidemiological studies. The assay may also support disease prevention and control in Chinese tongue sole aquaculture.

## 1. Introduction

*Cynoglossus semilaevis* occurs naturally in the coastal waters of Bohai Bay and the Yellow Sea. This fish has become an important marine aquaculture species in China. In recent years, emerging diseases have caused high mortality in farmed Chinese tongue soles and major economic losses. *Cynoglossus semilaevis* papillomavirus (CsPaV) is a recently identified virus associated with mortality rates of up to 80.7% [1]. However, no sensitive quantitative method is available for CsPaV detection. This lack of a suitable method has limited epidemiological studies and the development of disease-control measures.

Papillomaviruses (PVs) are linked to many diseases in humans and animals [2,3]. Researchers identified the first fish papillomavirus in gilthead seabream (*Sparus aurata*) in 2016 [4]. Researchers later reported fish PVs in *Lutjanus campechanus*, *Oncorhynchus mykiss* [5], *Silurus glanis* [6], *Trematomus bernacchii*, *Centropristis striata*, *Melanogrammus aeglefinus* [7], Tibetan cold-water fish [8], *Betta splendens* [9], *Cynoglossus semilaevis* [1], and *Raja clavata* [10]. Mammalian papillomaviruses have been studied in detail. In contrast, fish papillomaviruses remain poorly understood. Known fish PV genomes contain the conserved E1, E2, L1, and L2 genes, but they lack the common E6 and E7 oncogenes. Phylogenetic analyses place these viruses in a distinct fish-associated lineage [11,12]. Their host range, target cells, routes of transmission, persistence, and role in disease remain unclear. A quantitative assay specific to CsPaV is therefore needed to study its distribution and its association with disease in aquaculture.

Common methods for pathogen detection include enzyme-linked immunosorbent assay (ELISA) [13], polymerase chain reaction (PCR) [14], loop-mediated isothermal amplification (LAMP) [15], and quantitative PCR (qPCR) [16]. ELISA requires pathogen-specific antibodies and includes several handling steps. Conventional PCR and LAMP are simple and sensitive, but they usually do not measure pathogen quantity. TaqMan probe-based qPCR provides high specificity, high sensitivity, and real-time quantification in a closed tube. This design also reduces handling after amplification and lowers the risk of contamination. In recent years, researchers have developed TaqMan probe-based qPCR assays for several aquatic animal viruses. These viruses include megalocytiviruses [16], tilapia lake virus [17], tilapia parvovirus [18], and turbot circovirus [19]. These studies show that TaqMan probe-based qPCR is useful for the quantitative surveillance of aquatic viral diseases. However, no similar assay had been developed for CsPaV.

This study aimed to develop and validate a TaqMan probe-based qPCR assay for CsPaV detection. We also used the assay in a preliminary molecular epidemiological study. The assay provides a practical tool for routine surveillance, field screening, and studies of CsPaV distribution.

## 2. Materials and Methods

### 2.1. Sample Collection

Between 2023 and 2026, we collected 176 clinically diseased Chinese tongue soles from aquaculture farms in Tianjin, Hebei Province, and Shandong Province. We also collected wild Chinese tongue soles from Bohai Bay and the Yellow Sea. In addition, we obtained *Epinephelus* sp., *Charybdis japonica*, *Rapana venosa*, *Litopenaeus vannamei*, *Chlamys farreri*, *Oratosquilla oratoria*, *Lateolabrax japonicus*, *Scapharca subcrenata*, and *Synechogobius hasta* from a seafood market near Bohai Bay. Table 1 provides the sampling locations, dates, species, tissues, sample numbers, and body lengths. We used these samples for the epidemiological survey. We transported all specimens to the laboratory in insulated boxes under chilled conditions. We collected the liver, spleen, and kidney from each Chinese tongue sole under aseptic conditions. We collected the tissues listed in Table 1 from the other species. We stored all tissue samples at −80 °C until nucleic acid extraction. Laboratory stocks of viral nervous necrosis virus (VNNV), spring viraemia of carp virus (SVCV), grass carp reovirus (GCRV), *Vibrio harveyi*, *V. alginolyticus*, *V. anguillarum*, and *Edwardsiella tarda* served as non-target controls in the specificity assay.

### 2.2. Primers and TaqMan Probe Design

Based on the *CsPaV L1* gene sequence (GenBank accession no. OQ865369), a pair of primers and a TaqMan probe were designed using Primer Premier 5.0. (PREMIER Biosoft International, Palo Alto, CA, USA) Candidate primers were evaluated to minimize the formation of primer dimers and hairpin structures and to ensure specific amplification of a 99 bp region of the *L1* gene. The probe was designed within the amplified region, with a length of 20–40 nucleotides and a GC content of approximately 50%. Primers L1-a-F (5′-CGGAATTCCGATGCACTCTTCAAGGAA-3′) and L1-a-R (5′-CCCAAGCTTGGGTCAAACAAAAGTTGTAT-3′) were designed for amplifying the *L1* gene sequence with a length of 654 bp and constructing a standard plasmid. A pair of qPCR-specific primers was designed using the L1-a fragment sequence: qL1-a-F1 5′-AATCAGATCAAGAAGAAGACCCA-3′; qL1-a-R1 5′-CCTCATTATCATAGATGGTGCC-3′. The TaqMan probe sequence was designed as 5′-FAM-CTGGAACACCGAACAGTGACTATGGC-TAMRA-3′ for qPCR amplification, with the target fragment being 99 bp. All primers and the TaqMan probe were synthesized by Anshengda Biotechnology Co., Ltd. (Suzhou, China).

### 2.3. Plasmid and Standard Curve Construction

We amplified a 654-bp fragment of the *CsPaV L1* gene in a total volume of 25 µL. The reaction contained 2 µL of DNA template (134.6 ng/µL), 1 µL of an L1-a-F/L1-a-R primer mixture (10 µM), 2.5 µL of 10× TransTaq HiFi Buffer I (TransGen Biotech Co., Ltd., Beijing, China; Cat. No. AP131-01), 2 µL of dNTPs (2.5 mM), 0.25 µL of TransTaq HiFi DNA Polymerase (TransGen Biotech Co., Ltd., Beijing, China; Cat. No. AP131-01), and 17.25 µL of ddH_2_O. The thermal program started with denaturation at 94 °C for 5 min. The program then included 35 cycles at 94 °C for 30 s, 60 °C for 30 s, and 72 °C for 30 s. A final extension was performed at 72 °C for 5 min. We purified the PCR product with a gel extraction kit (TIANGEN Biotech (Beijing) Co., Ltd., Beijing, China; Cat. No. DP219). We then cloned the product into the pMD19-T vector (TaKaRa Bio Inc., Kusatsu, Shiga, Japan; Cat. No. 6013) according to the manufacturer’s instructions. We transformed the recombinant plasmid into competent *Escherichia coli* DH5α cells (Tsingke Biotechnology Co., Ltd., Beijing, China; Cat. No. DLC101). We confirmed positive clones by Sanger sequencing. We purified the plasmid with a plasmid extraction kit (TIANGEN Biotech (Beijing) Co., Ltd., Beijing, China; Cat. No. DP105). We measured the plasmid concentration with a NanoDrop spectrophotometer (Thermo Fisher Scientific, Waltham, MA, USA).

We quantified the recombinant plasmid as described previously [20]. We calculated its copy number using the formula reported in [21]. We prepared 10-fold serial dilutions of pMD19T-L1a from 5.8 × 10^10^ to 5.8 × 10^1^ copies/µL. We constructed the standard curve by plotting the Ct value against the base-10 logarithm of the plasmid copy number.

### 2.4. TaqMan Probe-Based qPCR Assay

The TaqMan probe-based qPCR reaction had a total volume of 20 µL. Each reaction contained 2 µL of template, 10 µL of Premix Ex Taq (TaKaRa Bio Inc., Shiga, Japan; Cat. No. RR390A), 0.8 µL of a qL1-a-F1/qL1-a-R1 primer mixture (10 µM each), 0.8 µL of TaqMan probe (10 µM), and 6.4 µL of ddH_2_O. We ran the assay on a QuantStudio 1 Plus Real-Time PCR System (Thermo Fisher Scientific, Waltham, MA, USA). The program started at 95 °C for 30 s. It then included 40 cycles at 95 °C for 5 s and 60 °C for 30 s. The program ended with a step at 50 °C for 30 s.

### 2.5. Sensitivity and Specificity Test

We prepared 10-fold serial dilutions of the L1-a recombinant plasmid. We used each dilution as a template for both qPCR and conventional PCR. The conventional PCR reaction had a total volume of 25 µL. Each reaction contained 2 µL of template, 22 µL of Fast Taq MasterMix (Beijing Biomed Gene Technology Co., Ltd., Beijing, China; Cat. No. MT302), and 1 µL of a qL1-a-F1/qL1-a-R1 primer mixture (10 µM each). The program started at 94 °C for 5 min. It then included 40 cycles at 94 °C for 10 s, 60 °C for 10 s, and 72 °C for 10 s. A final extension was performed at 72 °C for 15 min.

The SYBR Green qPCR reaction had a total volume of 20 µL. Each reaction contained 2 µL of template, 10 µL of 2× Universal SYBR Green qPCR Master Mix (Sangon Biotech (Shanghai) Co., Ltd., Shanghai, China; Cat. No. B690016), 0.8 µL of a qL1-a-F1/qL1-a-R1 primer mixture (10 µM each), and 7.2 µL of ddH_2_O. The program started at 95 °C for 2 min. It then included 40 cycles at 95 °C for 10 s and 60 °C for 30 s. The program ended with a step at 50 °C for 30 s.

We evaluated analytical specificity with SVCV, GCRV, VNNV, *Vibrio harveyi*, *V. alginolyticus*, *V. anguillarum*, and *Edwardsiella tarda*. These pathogens served only as non-target controls for cross-reactivity testing.

### 2.6. Reproducibility Test

We tested each plasmid concentration in three technical replicates within one run to assess intra-assay repeatability. We calculated the mean Ct value, standard deviation (SD), and coefficient of variation (CV) for each concentration. We repeated the assay after 7 days to assess inter-assay repeatability. We again calculated the mean Ct value, SD, and CV.

### 2.7. Infection Experiments

Thirty healthy Chinese tongue soles (mean body length: 25 ± 2 cm) were randomly assigned to an infection group or a control group. Before the challenge, the fish were acclimated for 7 days in static tanks with continuous aeration. Approximately one-third of the water was replaced daily. The water temperature was maintained at 21 ± 1 °C, and the salinity was maintained at 20 ± 1 ‰. The fish were fasted for 24 h before inoculation. Normal feeding resumed on the day after inoculation. The inoculum was prepared from a tissue homogenate obtained from diseased fish. The homogenate was filtered through a 0.45 µm membrane. Each fish in the infection group received an intraperitoneal injection of 300 µL of inoculum containing 3.66 × 10^5^ CsPaV copies/µL. Each control fish received an equal volume of sterile PBS. The fish were not anesthetized during inoculation. We recorded mortality and clinical signs daily. At 1, 2, 3, 5, and 7 days post-infection, we randomly sampled three fish from each group as independent biological replicates. We collected the liver, spleen, kidney, intestine, gill, muscle, heart, skin mucus, blood, and eye from each fish. We measured CsPaV DNA loads with the established TaqMan qPCR assay. We analyzed each DNA sample in three technical qPCR replicates.

### 2.8. Statistics Analysis

We performed all statistical analyses with OriginPro 8.0 (OriginLab Corporation, Northampton, MA, USA) and SPSS Statistics 24.0. (IBM Corp., Armonk, NY, USA) We constructed the standard curve by linear regression of Ct values against the base-10 logarithm of the plasmid copy number. We assessed linearity with the coefficient of determination (R^2^). We evaluated intra-assay and inter-assay repeatability with the mean Ct value, SD, and CV. We analyzed viral loads from different tissues of the same fish by one-way repeated-measures ANOVA followed by Bonferroni-adjusted comparisons. We compared adjacent sampling times with Welch’s *t*-test and applied the Holm–Bonferroni correction. We compared the three independent clinical-syndrome groups with Welch’s one-way ANOVA. We present the results as the mean ± SD. We considered *p* < 0.05 statistically significant.

### 2.9. Ethics Statement

The Institutional Animal Care and Use Committee of Tianjin Normal University approved all animal procedures (Approval/Registration No. 2024041001; approval date: 1 January 2024). We conducted all procedures according to applicable institutional and local regulations.

## 3. Results

### 3.1. Establishment of the Standard Curve for TaqMan Probe-Based qPCR

**Figure 1:**
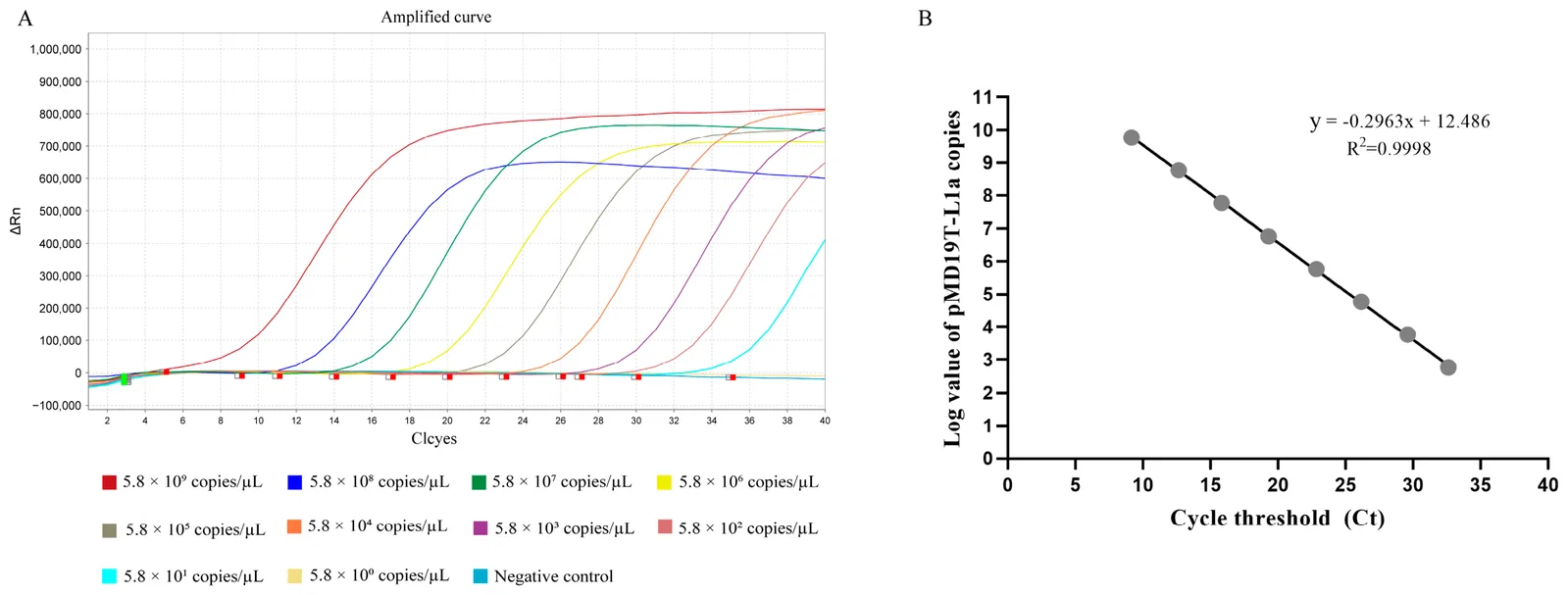
Establishment of a TaqMan probe-based qPCR assay for CsPaV detection. (**A**) Serial dilutions of the pMD19T-L1a plasmid produced the amplification curves. (**B**) The standard curve shows the relationship between Ct values and the base-10 logarithm of the pMD19T-L1a plasmid copy number.

The initial concentration of the recombinant plasmid was 214.3 ng/µL. We prepared 10-fold serial dilutions and used them as standards for TaqMan probe-based qPCR. The assay produced typical S-shaped amplification curves over a range from 5.8 × 10^10^ to 5.8 × 10^1^ copies/µL (Figure 1A). The Ct values increased as the template concentration decreased. Linear regression showed a strong linear relationship, with an R^2^ value of 0.9998 (Figure 1B). All intra-assay and inter-assay CVs were below 2% (Table 2). These results show that the assay has good repeatability and stability.

### 3.2. Sensitivity and Specificity of the TaqMan Probe-Based qPCR Assay

**Figure 2:**
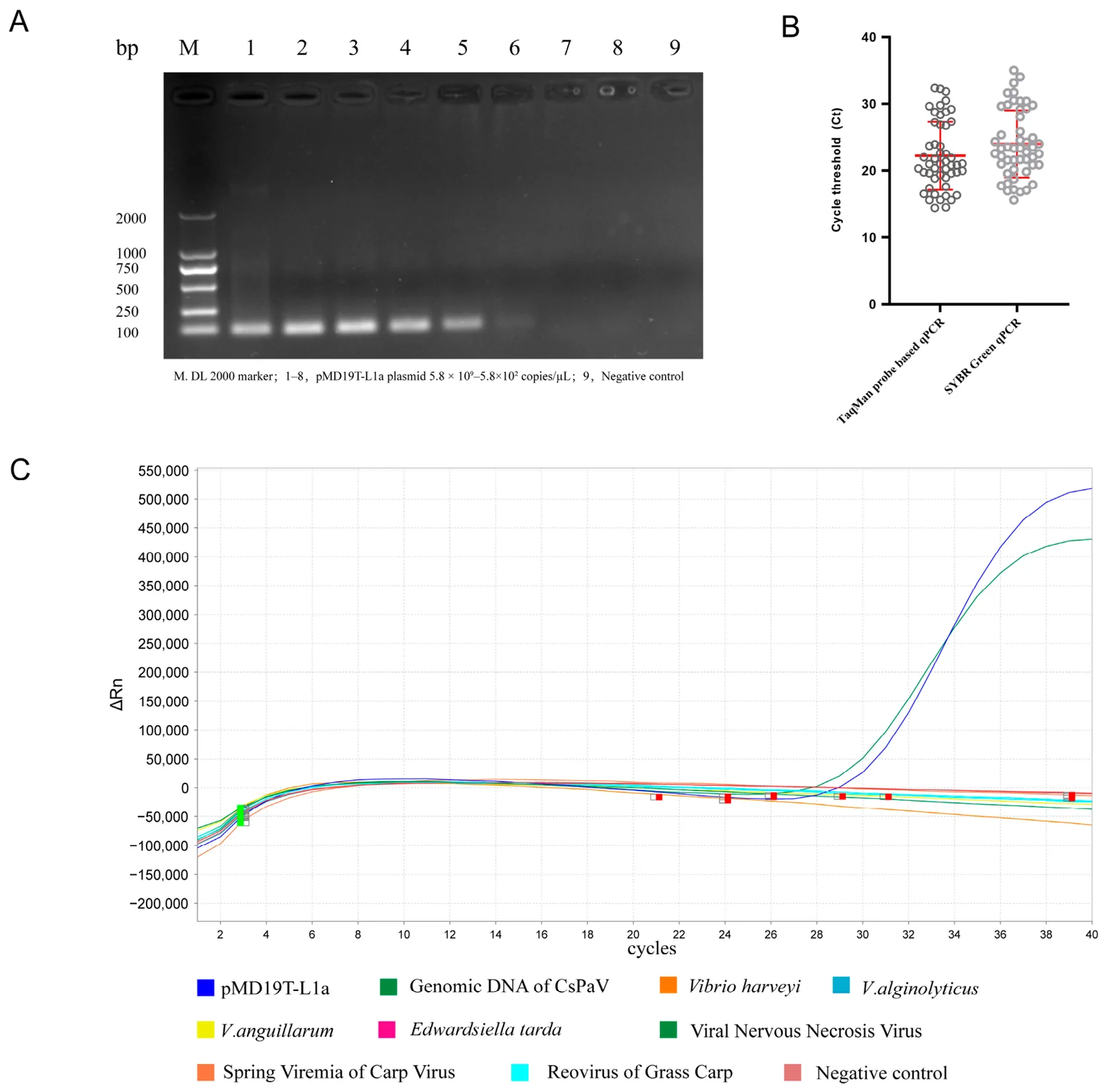
Evaluation of the sensitivity and specificity of the TaqMan probe-based qPCR assay. (**A**) Serially diluted pMD19T-L1a plasmids were tested by conventional PCR. (**B**) The sensitivities of the TaqMan probe-based qPCR and SYBR Green qPCR assays were compared. (**C**) The analytical specificity of the TaqMan probe-based qPCR assay was evaluated.

We prepared 10-fold serial dilutions of pMD19T-L1a and analyzed the amplification curves and Ct values at each concentration. The TaqMan probe-based qPCR assay detected as few as 5.8 × 10^1^ copies/µL. This detection limit was 1000-fold lower than that of conventional PCR (Figure 2A). We also tested 50 spleen samples from Chinese tongue soles with both the TaqMan probe-based qPCR assay and the SYBR Green qPCR assay. Both assays produced the same positive rate. However, the TaqMan assay produced lower Ct values than the SYBR Green assay (Figure 2B). This result indicated greater analytical sensitivity under the conditions tested.

Only pMD19T-L1a and CsPaV genomic DNA produced typical amplification curves (Figure 2C). The other aquatic pathogens produced no specific amplification. These results show that the assay is specific for CsPaV under the conditions tested.

### 3.3. Tissue Distribution and Proliferation Kinetics of CsPaV

**Figure 3:**
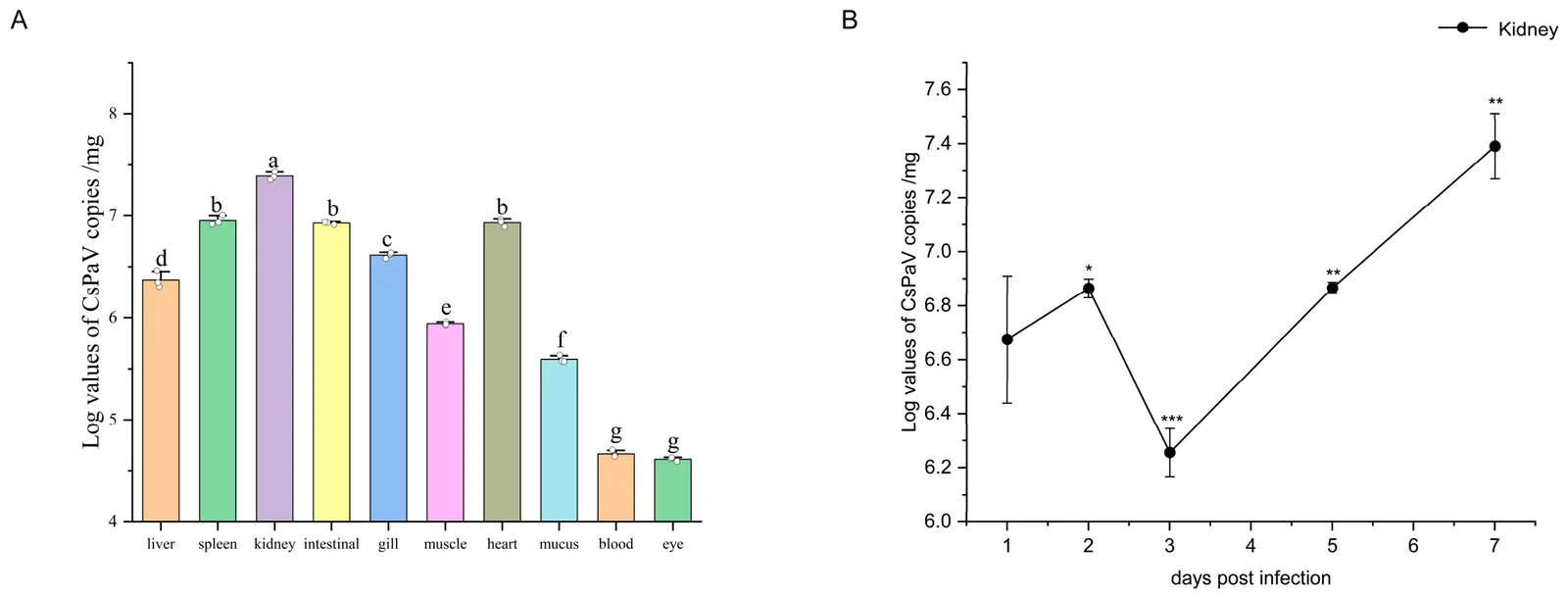
Tissue distribution and temporal dynamics of CsPaV after experimental infection in Chinese tongue soles. (**A**) The bars show CsPaV loads in tissues from experimentally infected fish. Different lowercase letters indicate significant differences among tissues. Bars with the same letter are not significantly different (*p* < 0.05). (**B**) The line shows temporal changes in CsPaV loads in kidney tissue after experimental infection. (Asterisks indicate statistically significant differences: * *p* < 0.05, ** *p* < 0.01, and *** *p* < 0.001.)

We used the established TaqMan probe-based qPCR assay to examine the tissue distribution and temporal changes in CsPaV DNA after experimental infection. The assay detected CsPaV DNA in all tissues examined. The kidney had the highest viral DNA load at 2.47 × 10^7^ copies/mg (Figure 3A). Viral DNA loads differed significantly among tissues (*p* < 0.05). Groups with the same lowercase letter did not differ significantly. The kidney viral DNA load changed over time. It decreased to 1.80 × 10^6^ copies/mg at 3 days post-infection and then increased to 2.47 × 10^7^ copies/mg at 7 days post-infection (Figure 3B).

### 3.4. Analysis of CsPaV Load in Fish with Different Disease Syndromes

**Figure 4:**
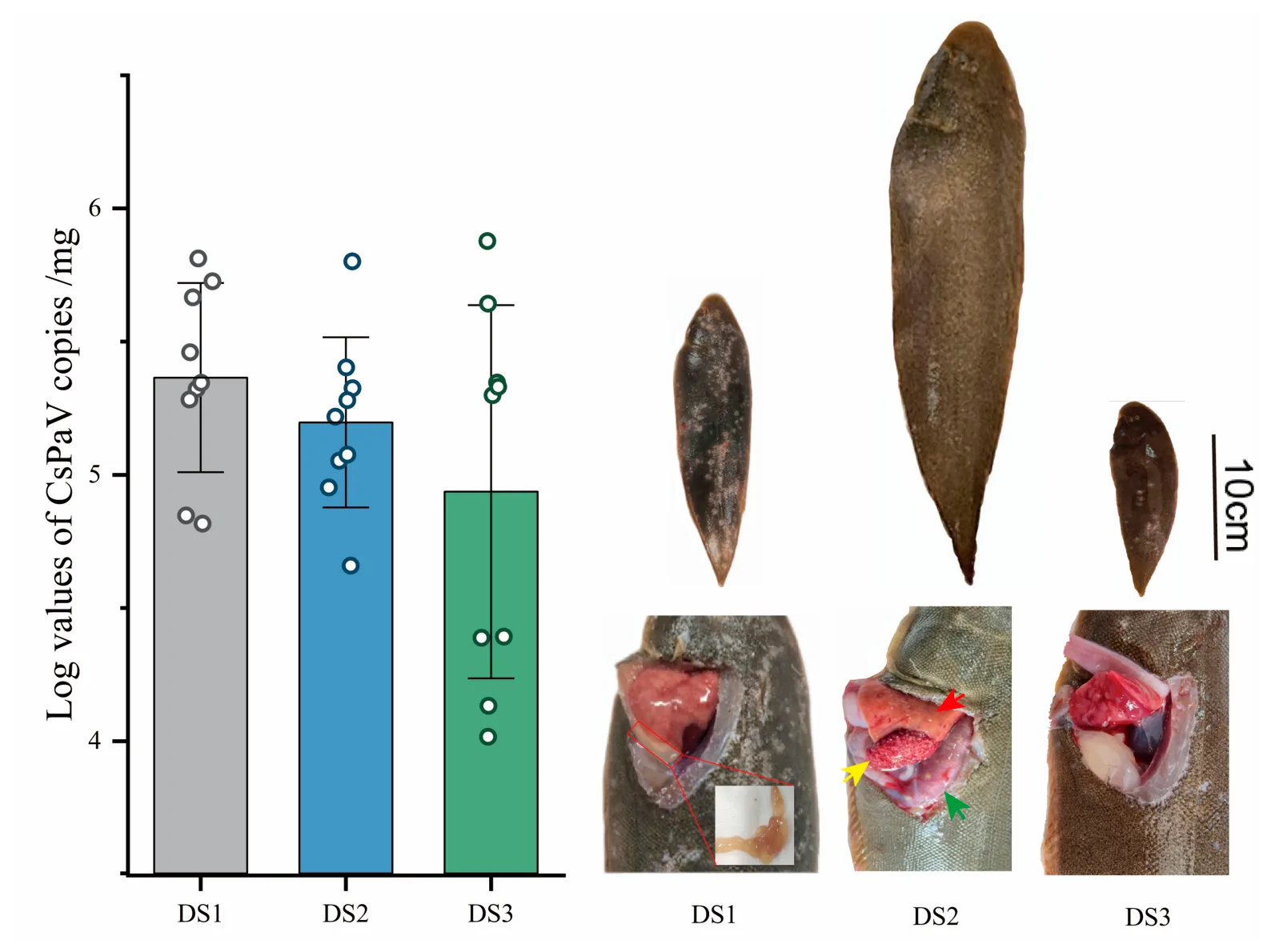
CsPaV loads in Chinese tongue soles with different clinical syndromes. DS1 fish were 25–30 cm long and showed extensive scale loss, scattered skin ulcers, and intestinal fluid accumulation. DS2 fish were 38–45 cm long and showed abdominal swelling with splenic and renal nodules. The red arrow indicates a pale, swollen liver with nodules. The yellow arrow indicates a swollen spleen with nodules. The green arrow indicates a pale, swollen kidney with nodules. DS3 fish were 15–20 cm long and showed skin ulcers without clear internal lesions.

We observed three clinical syndromes in CsPaV-positive Chinese tongue soles of different body sizes. DS1 fish were 25–30 cm long and showed extensive scale loss, scattered skin ulcers, and intestinal fluid accumulation. DS2 fish were 38–45 cm long and showed abdominal swelling with splenic and renal nodules. DS3 fish were 15–20 cm long and showed skin ulcers without clear internal lesions (Figure 4). The mean CsPaV DNA loads were 2.31 × 10^5^ copies/mg in DS1, 1.57 × 10^5^ copies/mg in DS2, and 8.64 × 10^4^ copies/mg in DS3. DS1 had the highest mean value, followed by DS2 and DS3. However, the three groups did not differ significantly (Welch’s one-way ANOVA, F(2, 15.05) = 1.42, *p* = 0.272).

### 3.5. Molecular Epidemiological Analysis of CsPaV in Chinese Tongue Soles and Other Species

**Figure 5:**
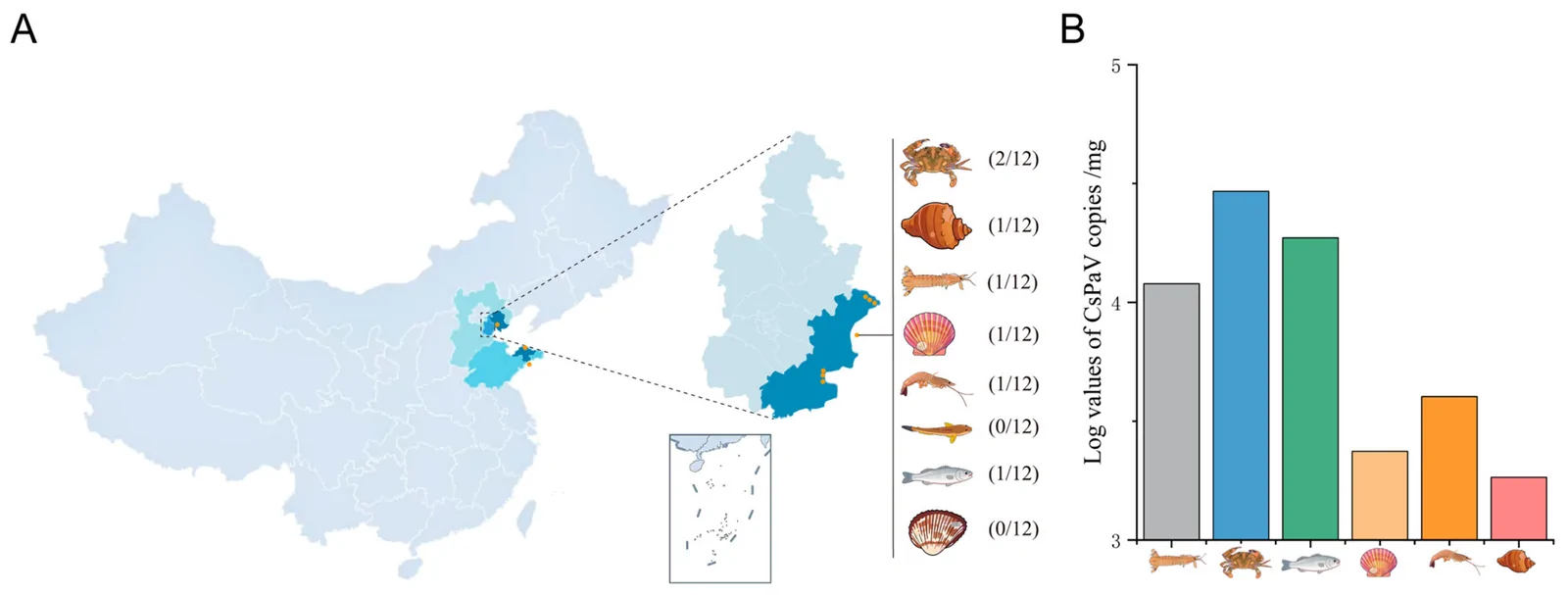
Epidemiological findings and detection of CsPaV in field samples. (**A**) The map shows the prevalence of CsPaV in different sampling regions. (**B**) The graph shows CsPaV detection rates and viral DNA loads in other aquatic species.

Between 2023 and 2026, we collected 176 Chinese tongue soles from Tianjin, Hebei Province, Shandong Province, Bohai Bay, and the Yellow Sea. The samples included juveniles about 5–8 cm long and adults longer than 15 cm. We tested kidney samples for CsPaV with the TaqMan probe-based qPCR assay. The overall positive rate was 80.68%. All CsPaV-positive Chinese tongue soles were adults (Figure 5A).

We screened 12 specimens from each of eight selected mollusk, crustacean, and fish species to examine the wider distribution of CsPaV DNA. The assay detected CsPaV DNA in six species. The positive results included *Charybdis japonica* (2/12, 16.7%), *Rapana venosa* (1/12, 8.3%), *Litopenaeus vannamei* (1/12, 8.3%), *Chlamys farreri* (1/12, 8.3%), *Oratosquilla oratoria* (1/12, 8.3%), and *Lateolabrax japonicus* (1/12, 8.3%) (Figure 5B). The assay did not detect CsPaV DNA in *Scapharca subcrenata* or *Synechogobius hasta*.

## 4. Discussion

Global aquaculture has grown rapidly and now provides a major part of the world’s food supply [22]. However, viral diseases in aquatic animals threaten the long-term development of this industry [23,24]. Emerging viruses are a particular concern. Researchers have recently reported several new viral diseases in fish. These diseases include scale drop disease in Asian sea bass caused by a megalocytivirus [25] and infections linked to previously unknown filoviruses, hantaviruses, and rhabdoviruses in European perch [26]. Other examples include koi sleepy disease caused by carp edema virus [27] and a possible new calicivirus genus detected in yellow catfish [28]. In 2024, our group first identified and described CsPaV in diseased Chinese tongue soles [1]. We proposed that CsPaV represents a new genus in the family Papillomaviridae [1]. Most emerging fish viruses lack commercial vaccines and specific antiviral treatments. Sensitive detection methods are therefore important for surveillance, early diagnosis, and timely disease control.

The major capsid protein L1 is produced at high levels and can assemble into virus-like particles that resemble native papillomavirus particles [29,30]. The L1 protein is abundant and strongly immunogenic. Its sequence also differs among virus types. These features have supported the use of L1 in papillomavirus detection and disease assessment [31,32]. The *L1* gene is therefore a suitable target for molecular detection. In this study, we designed CsPaV L1-specific primers and a hydrolysis probe. We then established a sensitive and specific TaqMan probe-based qPCR assay.

Our assay uses a sequence-specific hydrolysis probe and closed-tube quantification, as do other TaqMan assays for aquatic viruses. Previous studies have shown that non-lethal samples can support repeated surveillance of aquatic viral infections [33,34]. Skin mucus may also serve as a non-lethal sample for CsPaV testing. Mucus swabs could allow repeated sampling without sacrificing valuable broodstock. However, skin mucus contained less CsPaV DNA than kidney tissue in this study. Paired-sample studies must therefore compare mucus and kidney testing before mucus swabs are used in the field. These studies should assess diagnostic sensitivity, agreement between sample types, and suitable decision limits. The CsPaV L1 assay had a wide linear range, a detection limit of 5.8 × 10^1^ copies/µL, no cross-reactivity with the non-target pathogens tested, and low intra-assay and inter-assay CVs. However, these findings do not prove that the assay is clinically better than other methods. This study did not include direct comparisons between laboratories or a diagnostic-accuracy study.

Our previous study described tissue lesions associated with CsPaV infection [1]. However, the present viral DNA data cannot show whether tissue viral load is related to lesion severity. Future infection studies should combine repeated viral-load measurements with histopathology and in situ viral detection. These methods could show where CsPaV occurs in relation to tissue lesions. In the present study, we detected CsPaV DNA in the liver, spleen, kidney, intestine, muscle, heart, gill, skin mucus, blood, and eye. This result indicates a wide distribution of viral DNA. The kidney had the highest viral DNA load, whereas the eye had the lowest load. The kidney is a major blood-forming and immune organ in fish. The high DNA load may reflect viral accumulation or tissue preference. However, assays for viral RNA or antigen are needed to confirm active replication in the kidney. Other important fish viruses, including lymphocystis disease virus [35] and infectious salmon anemia virus [36], also target the kidney or use it as a main diagnostic tissue. Our results support kidney tissue as the preferred sample for clinical detection. Skin mucus had a lower viral DNA load, but all mucus samples tested positive. Skin mucus may therefore be useful for non-lethal sampling. Larger studies with repeated sampling must confirm this possibility.

CsPaV-positive Chinese tongue soles showed different clinical signs across body-size groups. However, the viral DNA loads did not differ significantly among the three syndrome groups. DS1 fish had the highest mean viral DNA load and showed scale loss, skin ulcers, and intestinal fluid accumulation. DS2 fish had an intermediate mean load and showed abdominal swelling, enlarged spleens and kidneys, and many white nodules. DS3 fish had the lowest mean load and showed mainly surface ulcers. These patterns may help guide future studies, but they do not show that body size caused the observed differences. Intestinal fluid accumulation may involve the intestinal lining or disturbed salt and water balance. Histopathology and in situ viral detection are needed to identify the affected cells. The lesions in DS2 resemble those reported in fish infected with *Mycobacterium* spp. [37] or red seabream iridovirus [38]. Researchers must exclude other infections before these lesions can be linked to CsPaV. Age-matched infection studies and immune-response measurements are also needed to test whether fish development affects disease outcome.

To investigate the potential environmental distribution and host range of CsPaV, we screened 12 specimens representing eight molluscans, other invertebrate, and fish species collected from Bohai Bay. CsPaV DNA was detected in one or two specimens from each of six species. Given the small sample size and the use of TaqMan probe-based qPCR alone, these detections should not be interpreted as evidence of productive infection or cross-class transmission; they may instead reflect transient exposure, environmental contamination, or mechanical carriage. Filter-feeding mollusks and other aquatic invertebrates may nevertheless serve as sentinels for the presence or enrichment of viral material in aquatic environments. Future studies should include larger, geographically and temporally stratified sample sets and should combine viral isolation, transcript-specific assays, and in situ hybridization to determine whether CsPaV enters and replicates in non-target hosts. Such evidence will be necessary to define the ecological reservoirs, transmission pathways, and aquaculture risks associated with CsPaV. This study has several limitations. The assay has not yet been comprehensively validated against genetically diverse CsPaV variants collected from different geographical regions and host species. Preliminary sequence analyses suggest that CsPaV may exhibit sequence variation among positive samples from different aquatic species, although these observations require further confirmation. Because mutations within the primer- or probe-binding regions could affect amplification efficiency and potentially lead to false-negative results, future studies should evaluate the conservation of these regions using larger, geographically representative sample sets and directly assess the effect of sequence variation on assay performance.

Farm monitoring programs should prioritize kidney tissue when they test dying or recently dead fish. Kidney tissue had the highest viral DNA load in this study. Routine monitoring could also include regular screening of healthy-looking broodstock and juveniles. Farm staff should promptly test fish that show scale loss, ulcers, abdominal swelling, or unusual mortality. Positive qPCR results in mollusks or crustaceans should be viewed as evidence of viral DNA in the environment or possible mechanical carriage. They should not yet be viewed as evidence that these animals are biological vectors. Confirmed positive results should lead to wider stock screening, movement control, equipment disinfection, and follow-up testing. Commercial farm studies are still needed to define action thresholds.

This study has several limitations. The infection experiment used only one dose, and the time-course analysis focused on the kidney. The data therefore cannot show a dose–response relationship or compare temporal patterns among different tissues. The assay measures CsPaV DNA, but it cannot separate inactive or remaining DNA from active viral replication. Viral RNA detection, antigen testing, in situ hybridization, or virus isolation is needed to confirm active infection. The assay has also not been fully tested against genetically diverse CsPaV variants from different regions and host species. Preliminary sequencing of positive samples from five aquatic species suggested sequence variation. However, these findings require further confirmation and are not presented as validated results. Changes in the primer- or probe-binding sites could reduce assay performance or cause false-negative results. Future studies should use larger and geographically diverse sample sets to test the conservation of these sites and the reliability of the assay.

## 5. Conclusions

In conclusion, we developed a sensitive and specific TaqMan probe-based qPCR assay that targets the *CsPaV L1* gene. The assay detected as few as 5.8 × 10^1^ copies/µL. It did not cross-react with the aquatic pathogens tested, and it showed good repeatability. The preliminary application identified kidney as the most suitable tissue among those examined. The assay also described temporal changes in kidney viral DNA after experimental infection. In addition, the assay supported screening of clinically diseased Chinese tongue soles and other aquatic species. The assay therefore provides a useful tool for CsPaV research and surveillance. However, the detection of viral DNA alone does not confirm active infection, host competence, or viral transmission. Further studies should include samples from different regions and genetic groups. These studies should also compare paired non-lethal samples and use methods that detect viral transcripts or locate viral material in tissues. Such work is needed before researchers can define standard diagnostic thresholds and farm-level disease-control procedures.

## Figures and Tables

**Table 1:** Sampling information and CsPaV detection results for Chinese tongue soles and other aquatic species collected between 2023 and 2026.

Province/City/Sea Area of Sample Collection	Collection Time	Species	Target Tissues	Collection Quantity	CsPaV-Positive Rate	Body Length
Tianjin	January 2023	*Cynoglossus semilaevis*	L, S, K	9	100%	15–20 cm; 40–45 cm
	March 2023		L, S, K	35	100%	15–25 cm; 35 cm; 40–45 cm; 50–55 cm
	April 2023		L, S, K	7	100%	15–20 cm
	May 2023		L, S, K	10	100%	15–20 cm
	July 2023		L, S, K	6	100%	35–45 cm
	September 2023		L, S, K	5	100%	35–45 cm
	September 2023		L, S, K	17	100%	15–20 cm; 35–45 cm
	April 2024		L, S, K	9	100%	35–45 cm
	April 2026		L, S, K	7	57%	5–8 cm; 45–50 cm
Hebei/Tangshan	May 2023		L, S, K	10	100%	15–20 cm
	March 2025		L, S, K	30	0	3–5 cm
Shandong/Yantai	May 2023		L, S, K	13	100%	10–15 cm
Bohai sea/117.59–118.00° E, 39.08–39.10° N	October 2023		L, S, K	5	100%	15–25 cm
Yellow sea/Waters off Renjiatai, Qingdao (120.48° E, 36.097243° N)	April 2024		L, S, K	13	100%	15–20 cm
Tianjin *	December 2024	*Epinephelus* sp.	L, S, K	10	100%	20–25 cm
	September 2025	*Rapana venosa*	M	12	8.3%	8–10 cm
		*Charybdis Japonica*	M	12	16.7%	15–20 cm
		*Litopenaeus vannamei*	H	12	8.3%	15–20 cm
		*Chlamys farreri*	M	12	8.3%	8–10 cm
		*Scapharca subcrenata*	M	12	0	3–5 cm
		*Oratosquilla oratoria*	M	12	8.3%	13–15 cm
		*Lateolabrax japonicus*	L	12	8.3%	18–23 cm
		*Synechogobius hasta*	L	12	0	15–18 cm
	April 2026	*Epinephelus* sp.	L, S, K	3	0	15–20 cm

Note: * indicates specimens obtained from coastal seafood markets in Tianjin. All products sold at these markets originated from Bohai Bay. Abbreviations: L, liver; S, spleen; K, kidney; M, muscle; H, hepatopancreas.

**Table 2:** Intra-assay and inter-assay repeatability of the CsPaV TaqMan probe-based qPCR assay.

Repeatability	Plasmid Concentration (Copies/µL)	Ct Value					
		Repeat 1	Repeat 2	Repeat 3	Mean	SD	CV (%)
Inter-group	10^8^	12.7	12.6	12.594	12.631	0.06	0.47
	10^7^	15.866	15.798	15.779	15.814	0.05	0.29
	10^6^	19.195	19.27	19.419	19.294	0.11	0.59
	10^5^	22.955	22.941	22.615	22.837	0.19	0.84
	10^4^	26.125	25.209	26.095	26.143	0.06	0.23
Intra-group	10^8^	12.631	12.702	12.56	12.631	0.07	0.56
	10^7^	15.814	15.963	15.562	15.78	0.2	1.29
	10^6^	19.294	18.738	18.954	18.995	0.28	1.48
	10^5^	22.837	22.756	22.685	22.685	0.2	0.87
	10^4^	26.143	25.931	25.665	25.706	0.24	0.92

## Data Availability

The authors confirm that the data supporting the findings of this study are fully available within the article.
